# Interactions of miR-323/miR-326/miR-329 and miR-130a/miR-155/miR-210 as prognostic indicators for clinical outcome of glioblastoma patients

**DOI:** 10.1186/1479-5876-11-10

**Published:** 2013-01-09

**Authors:** Shuwei Qiu, Sheng Lin, Dan Hu, Yimin Feng, Yang Tan, Ying Peng

**Affiliations:** 1 Department of Neurology, The Sun Yat-sen Memorial Hospital, Sun Yat-sen University, Guangzhou, 510120, China; 2 Department of Internal Medicine, College of Medicine, East Tennessee State University, Johnson City, 37604, TN, USA; 3 Laboratory of Medical Genetics, Key Laboratory of Birth Defects Surveillance and Intervention of Guangdong Province, Shenzhen Research Institute of Population and Family Planning, Shenzhen, 518040, China; 4 Department of Neurology, Renmin Hospital of Wuhan University, Wuhan, 430060, China

**Keywords:** Glioblastoma multiforme, microRNA, Prognostic marker, Overall survival, Progression-free survival, Interaction

## Abstract

**Background:**

Glioblastoma multiforme (GBM) is the most common and aggressive brain tumor with poor clinical outcome. Identification and development of new markers could be beneficial for the diagnosis and prognosis of GBM patients. Deregulation of microRNAs (miRNAs or miRs) is involved in GBM. Therefore, we attempted to identify and develop specific miRNAs as prognostic and predictive markers for GBM patient survival.

**Methods:**

Expression profiles of miRNAs and genes and the corresponding clinical information of 480 GBM samples from The Cancer Genome Atlas (TCGA) dataset were downloaded and interested miRNAs were identified. Patients’ overall survival (OS) and progression-free survival (PFS) associated with interested miRNAs and miRNA-interactions were performed by Kaplan-Meier survival analysis. The impacts of miRNA expressions and miRNA-interactions on survival were evaluated by Cox proportional hazard regression model. Biological processes and network of putative and validated targets of miRNAs were analyzed by bioinformatics.

**Results:**

In this study, 6 interested miRNAs were identified. Survival analysis showed that high levels of miR-326/miR-130a and low levels of miR-323/miR-329/miR-155/miR-210 were significantly associated with long OS of GBM patients, and also showed that high miR-326/miR-130a and low miR-155/miR-210 were related with extended PFS. Moreover, miRNA-323 and miRNA-329 were found to be increased in patients with no-recurrence or long time to progression (TTP). More notably, our analysis revealed miRNA-interactions were more specific and accurate to discriminate and predict OS and PFS. This interaction stratified OS and PFS related with different miRNA levels more detailed, and could obtain longer span of mean survival in comparison to that of one single miRNA. Moreover, miR-326, miR-130a, miR-155, miR-210 and 4 miRNA-interactions were confirmed for the first time as independent predictors for survival by Cox regression model together with clinicopathological factors: Age, Gender and Recurrence. Plus, the availability and rationality of the miRNA-interaction as predictors for survival were further supported by analysis of network, biological processes, KEGG pathway and correlation analysis with gene markers.

**Conclusions:**

Our results demonstrates that miR-326, miR-130a, miR-155, miR-210 and the 4 miRNA-interactions could serve as prognostic and predictive markers for survival of GBM patients, suggesting a potential application in improvement of prognostic tools and treatments.

## Background

Glioblastoma multiforme (GBM) is the most common and aggressive primary adult brain tumor. Despite advances in treatment modalities, the prognosis of GBM patients is very poor [1]. Therefore, it is urgent to develop new diagnostic and prognostic tools and treatments which may be beneficial for improving the clinical management of GBM. Currently, tumor stratifications relying on molecular profiles are increasingly prevalent and important. Furthermore, molecular and genetic profiling studies have identified several prognostic and predictive markers for GBM [2,3].

MicroRNAs (miRNAs or miRs), which are endogenous non-coding small RNAs, post-transcriptionally regulate gene expression through inhibition of translation or degradation of target mRNAs [4]. MiRNAs are aberrantly expressed in a variety of tumor types and exert important regulations on tumor biology via acting as oncogenes or tumor suppressors [5]. Recently, several studies indicate that expressions of miRNAs are associated with patients’ survival and are able to function as prognostic and predictive indicators [6,7]. Moreover, it has been confirmed that miRNA expression profiles are more accurate to classify tumors than mRNA profiles [8]. However, in the selection of miRNA markers for GBM prognosis, the applying of following aspects, such as small dataset, explanatory variables, single miRNA analysis, pre-selection of miRNAs and use of approaches, finally lead to a variety set of different miRNA markers.

The main purpose of this study is to identify specific miRNA markers that are closely associated with tumor progression and survivals for GBM patients by analyzing significantly altered miRNAs in a large dataset. Another goal is to investigate the availability and rationality of interactions of interested miRNAs as prognostic and predictive indictors for clinical outcome of GBM patients. In this study, we found that miR-326, miR-130a, miR-155, miR-210 and 4 miRNA-interactions could function as prognostic and predictive markers for survival of GBM patient.

## Materials and methods

### TCGA miRNA dataset and patient information

Expression data of miRNAs and genes and the corresponding clinical data for glioblastoma patients were downloaded from The Cancer Genome Atlas data portal (July 2012) [9]. 480 GBM patients with full annotation of Age, Gender, Survival time, Vital status, Time to progression/recurrence and miRNA values were identified in this study. There were 186 females and 294 male patients with ages 56.7 ± 15.9 and 58.1 ±13.6 years, respectively. Among the whole set, 318 patients suffered from tumor recurrence while 162 patients were kept away from progression/recurrence. Besides, miRNA expression data from 10 normal brain tissues (NBT) were also collected. The collection of the original material and data of TCGA was conducted in compliance with all applicable laws, regulations and policies for the protection of human subjects, and necessary IRB approvals were obtained [9]. Totally, Expression levels of 534 human microRNAs were detected using the Agilent 8 × 15K Human microRNA platform. The data was quantile-normalized, collapsed within miRNAs, and log2 transformed.

### Analysis of expression levels of miRNA data in GBM samples

To reflect the real expression profiles of miRNAs in GBM and normal brain tissues, and to yield the detection error, we analyzed three separate batches, batch 5, batch 16 and batch 20, from TCGA dataset with the biggest numbers of patients, incorporating 63, 47 and 46 patient samples respectively. The level 3 data were directly used to evaluate the relative expression levels of miRNAs in each sample by the Z-score method [10]. The mean Z-score of each miRNA from GBM and NBT samples was calculated and sorted in each batch according to Z-score values in GBM samples, and the top 20 most down-regulated and up-regulated miRNAs were illustrated in the heatmaps pattern. Only those microRNAs overlapping for two or three times among three batches were selected.

### Statistical computing methods

MiRNA expression profiles related to glioblastoma survival were identified using the Kaplan-Meier survival analysis and statistical significances of overall survival (OS) and Progression-Free survival (PFS) were determined using the Log-Rank test. Survival analysis was performed on SPSS (version 17.0; SPSS Inc.) and the survival curve was generated by GraphPad Prism (version 5.04; GraphPad Software, Inc.). For survival analysis, patients with survival time lesser than 30 days were excluded, since these patients might have died for reasons other than the disease itself. A total of 458 patients fitting this criterion were included for survival analysis. For stratification analysis of survival, expression levels of down-regulated miRNAs were sorted by ascending order, while the up-regulated miRNAs were sorted by descending order. Then, quartiles of 25%, 50% and 75% of the sorted miRNA values were set as cutoffs for low/high expressions of each miRNA. The survival time was expressed as mean ± SE. To determine whether expression levels of miRNAs were associated with Time to Progression (TTP), discrepancies of miRNA levels between groups were tested by student’s t-test, in terms of TTP of 9, 12 and 15 months. Likewise, according to whether there was recurrence or not, differences of miRNA levels between recurrent and non-recurrent groups were also tested using student’s t-test.

Next, the Cox proportional hazard regression model was used to determine the influences of miRNA expressions as well as clinicopathological factors (age, gender and recurrence) on patient survival. To adjust this potential effect that may be confounded by age, gender and recurrence, a multivariate Cox proportional hazard regression analysis using all these clinicopathological factors was performed. For analysis of interactions of two miRNAs, different quartile stratifications of the expression levels of miRNAs were set as cut-offs for high and/or low levels. The meaning of interaction in this text was defined as combined effect or coaction of high and/or low levels of two different miRNAs from one patient, e.g. high/low level of miR-A and high/low level of miR-B.

Functional Gene Ontology (GO) biological processes terms of the putative targets of candidate miRNAs were performed and the GO enrichment scores of target genes were presented in the form of heatmaps [11,12]. Putative targets of miRNAs were predicted by TargetScan Human 6.2 software [13]. Then the Functional Gene Ontology (GO) biological processes terms of the putative targets of candidate miRNAs, as well as the statistical analysis, were performed by DAVID Bioinformatics Resources 6.7 [14]. The network of validated targets of interested miRNAs was created by BisoGenet software, showing the interactions of validated targets [15]. The validated targets of interested miRNAs were obtained from Diana TarBase v6.0 [16] and miRecords [17] and extensive Pubmed publications search. The differences were considered statistically significant at p < 0.05.

## Results

### Screening of the most altered miRNAs expressed in GBM samples

**Figure 1:**
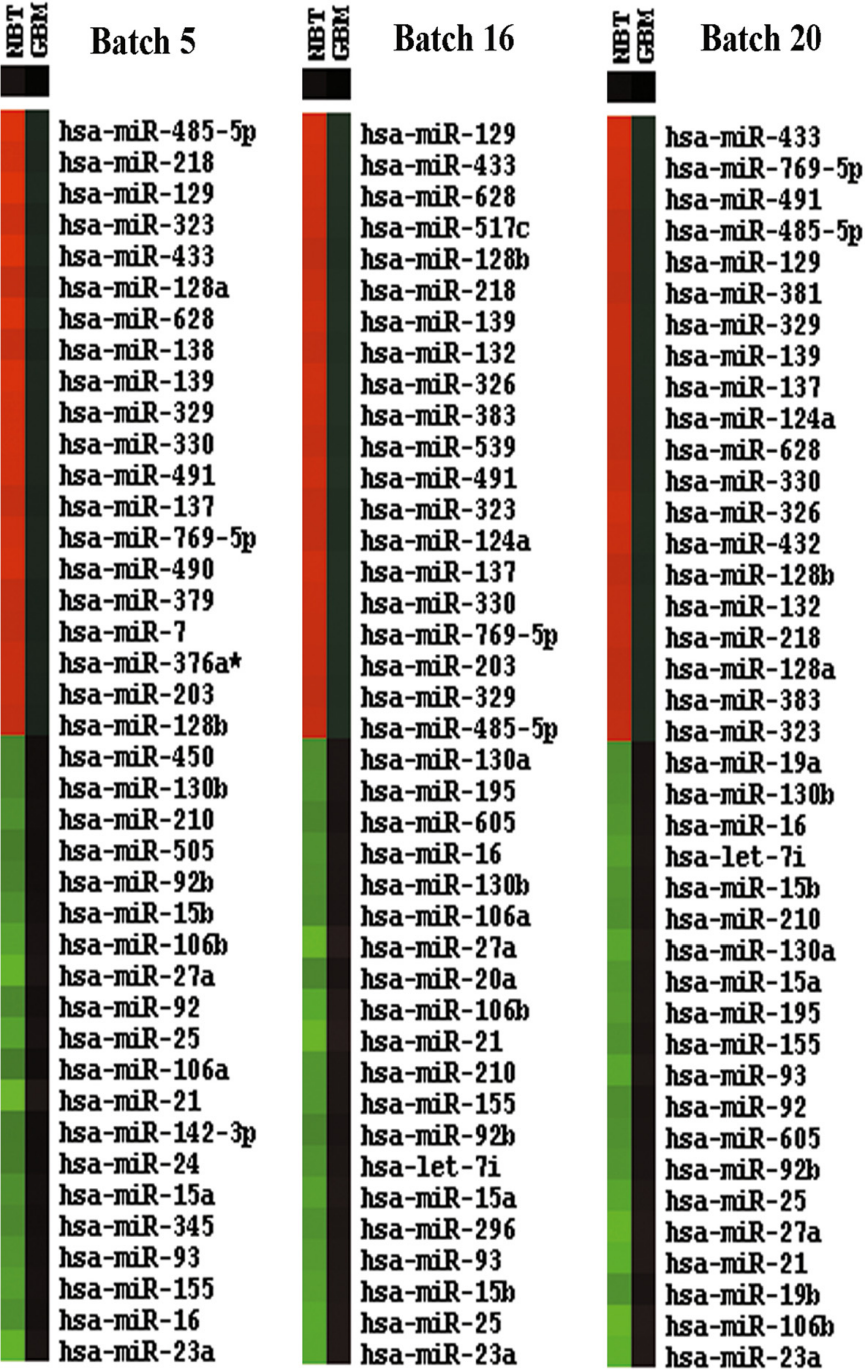
**Screening of miRNAs differentially expressed in GBM samples.** Shown are top 20 most down-regulated and up-regulated miRNAs in GBM samples within batch 5, 16 and 20 from TCGA dataset. MiRNAs overlapped no less than two times cross 3 batches are selected as candidates for further studies. Red denotes low levels of miRNAs in GBM samples, while green denotes low levels of miRNAs in normal brain tissues.

To decrease the detection error within and cross microarrays, mean Z-score values of each miRNA in bath5, 16 and 20, which incorporate the biggest numbers of GBM samples in TCGA dataset, were calculated and sorted, and then the top 20 most altered miRNAs in GBM samples were illustrated in the form of heatmaps. After overlap analysis, expression levels of 19 miRNAs were found to be decreased in GBM sam-ples, including miR-124a, miR-128a, miR-128b, miR-129, miR-132, miR-137, miR-139, miR-203, miR-218, miR-323, miR-326, miR-329, miR-330, miR-383, miR-433, miR-485-5p, miR-491, miR-628, and miR-769-5p, while 19 miRNAs increased in GBM samples, involving let-7i, miR-106a, miR-106b, miR-130a, miR-130b, miR-155, miR-15a, miR-15b, miR-16, miR-195, miR-21, miR-210, miR-23a, miR-25, miR-27a, miR-605, miR-92, miR-92b, and miR-93 (Figure 1). Expression levels of all these candidate miRNAs were validated by other independent miRNAs detection studies with RNA sequencing, real-time PCR or microarray [18-22]. 

### Correlations between miRNA expression and survival of GBM patients

**Figure 2:**
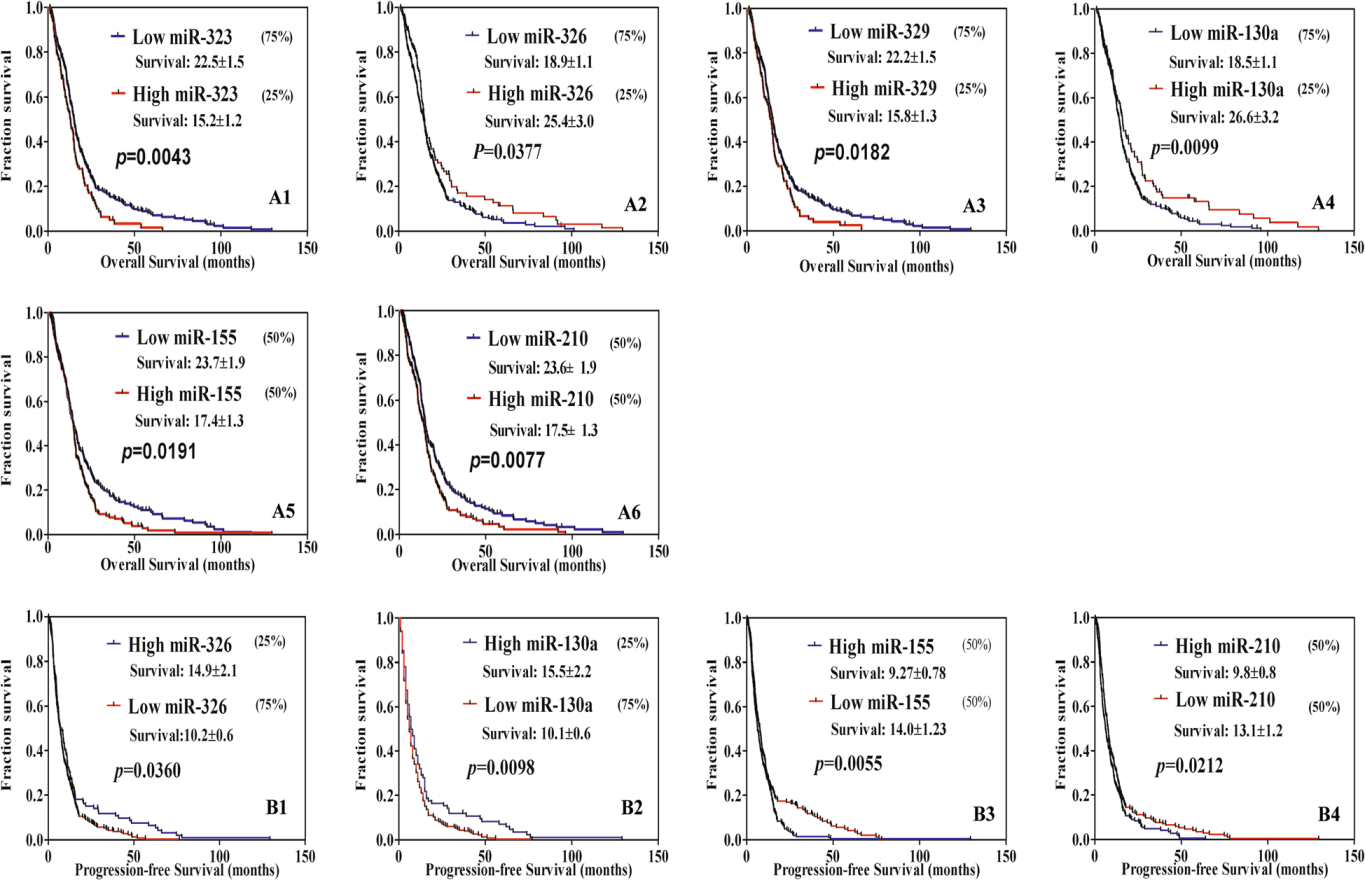
**Kaplan-Meier survival curves of the interested miRNAs in GBM patients. A**. Analysis of the interested miRNAs on patients’ Overall survival (OS). **B**. Analysis of the interested miRNAs on Progression-free survival (PFS). Survival time displayed here is mean ± SE.

Next, using Kaplan-Meier survival analysis, correlations between miRNA expression profiles and patient survival were performed. Expression levels of the 38 aberrantly expressed miRNAs were stratified by quartiles 25%, 50% and 75%. Totally, 16 miRNAs were shown to correlate with OS of GBM patients at different quartile stratifications (Additional file 1: Table S1). Only miR-323, miR-326, miR-329, miR-130a, miR-155 and miR-210 were selected as interested candidates for further analysis; this was because, according to our knowledge, no publications on patients survival associated with these miRNAs were reported. Our analysis showed that low expression levels of miR-323, miR-329, miR-155 and miR-210 significantly correlated with long OS survival (p = 0.0043, 0.0182, 0.0191 and 0.0077, respectively), while high expression levels of miR-326 and miR-130a were associated with long OS survival (p = 0.0377 and 0.0099; Figure 2A). We also showed that high levels of miR-326 and miR-130a were associated with extended survival without tumor progression (p = 0.036 and 0.0098; Figure 2B), while low expression levels of miR-155 and miR-210 were related with long PFS survival (p = 0.0055 and 0.0212; Figure 2B). These results suggested that miR-326 and miR-130a may serve as tumor suppressors, while miR-323, miR-329, miR-155 and miR-210 serve as oncogenes.

### The association among miRNAs expression and recurrence and time to progression in GBM patients

Both tumor recurrence and time to progression (TTP) are closely related to clinical outcome. Therefore, miRNA expression levels were evaluated to identify the differently expressed miRNAs according to tumor recurrence as well as long/short time to progression. Initially, we found that, among the 38 aberrant miRNAs, levels of miR-323 and miR-329 from the non-recurrent group (n = 162) were significantly increased in comparison to the recurrent group (n = 318; p = 0.0314 and 0.0364, respectively), whereas miR-132, miR-433, miR-628 and miR-769-5p were marginally increased in the non-recurrent group with miR-92b marginally decreased (Table 1). Then we analyzed expression discrepancies of miRNAs between long and short TTP groups. The results showed that significant up-regulations of miR-128b and miR-323 were observed in the group of TTP longer than 15 months (p = 0.0420 and 0.0451), with marginal increase of miR-329 level (p = 0.0981; Table 2). Likewise, among groups of TTP more than 9, 12 or 15months, obvious elevations of miR-106a, miR-106b and miR-92 were exhibited (p < 0.05), while levels of miR-21 was significantly reduced in the group of TTP more than 9 and 12months (p < 0.01; Table 2). However, no significant associations were observed between expression levels of miR-326/miR-130/miR-155/miR-210 and recurrence/TTP time.

**Table 1:** Differential expression levels of microRNAs associated with progression/recurrence or not in GBM patients

**microRNAs**	**Progression or Recurrence**		**p-value**
	**Yes**	**No**	
hsa-miR-132	7.82 ± 0.51	7.92 ± 0.49	0.0605
hsa-miR-323	6.19 ± 0.29	6.26 ± 0.41	0.0314
hsa-miR-329	6.06 ± 0.20	6.11 ± 0.32	0.0364
hsa-miR-433	5.95 ± 0.16	5.98 ± 0.27	0.0739
hsa-miR-628	6.31 ± 0.19	6.34 ± 0.21	0.0843
hsa-miR-769-5p	6.48 ± 0.23	6.53 ± 0.36	0.0553
hsa-miR-92b	8.91 ± 0.67	8.28 ± 0.69	0.0682

**Table 2:** Differential expression levels of microRNAs associated with time to progression in patients with GBM

**microRNAs**	**Time To Progression(TTP)**								
	**< 9 months**	**> 9 months**	**p-value**	**< 12 months**	**> 12 months**	**p-value**	**< 15 months**	**> 15 months**	**p-value**
hsa-miR-128b	7.56 ± 0.76	7.59 ± 0.74	0.6587	7.54 ± 0.75	7.67 ± 0.76	0.2028	7.53 ± 0.73	7.76 ± 0.81	0.0420
hsa-miR-323	6.19 ± 0.29	6.20 ± 0.28	0.5988	6.18 ± 0.29	6.22 ± 0.28	0.3012	6.18 ± 0.29	6.26 ± 0.30	0.0451
hsa-miR-329	6.06 ± 0.19	6.07 ± 0.21	0.6787	6.05 ± 0.20	6.07 ± 0.21	0.4293	6.05 ± 0.19	6.10 ± 0.22	0.0981
hsa-miR-106a	9.77 ± 0.77	9.95 ± 0.67	0.0439	9.77 ± 0.75	10.03 ± 0.67	0.0091	9.79 ± 0.73	10.03 ± 0.75	0.0305
hsa-miR-106b	10.69 ± 0.67	10.93 ± 0.58	0.0021	10.73 ± 0.67	10.91 ± 0.56	0.0398	10.75 ± 0.65	10.88 ± 0.60	0.1980
hsa-miR-21	14.22 ± 1.11	14.01 ± 1.04	0.1136	14.23 ± 1.07	13.86 ± 1.09	0.0089	14.23 ± 1.07	13.74 ± 1.08	0.0029
hsa-miR-92	10.15 ± 0.62	10.28 ± 0.64	0.0795	10.16 ± 0.62	10.32 ± 0.65	0.0497	10.16 ± 0.60	10.38 ± 0.71	0.0214

### The association between interactions of miRNAs and survival of GBM patients

**Figure 3:**
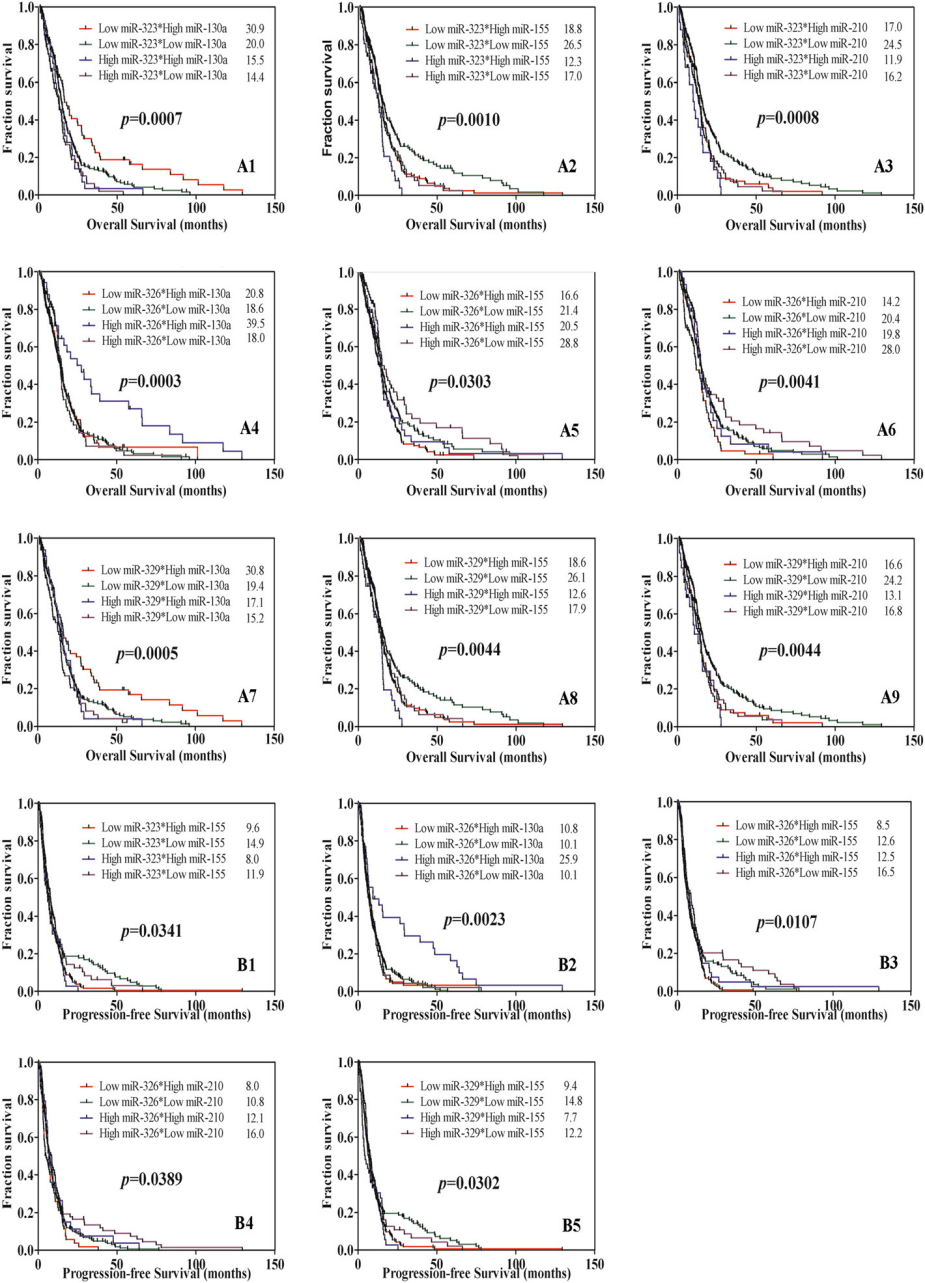
**Kaplan-Meier survival analysis of the miRNA-interactions. A**. Analysis of the miRNA-interaction on patients’ OS. **B**. Analysis of the miRNA-interaction on patients’ PFS.

As shown above, Low levels of miR-323 and miR-329 were associated with long survival, whereas high levels of both miRNA were present in patients without recurrence or long TTP time. This inconsistence highlighted the complication and importance of these two miRNAs in GBM tumor progression. Moreover, interactions of miRNAs may be more specific and feasible to discriminate and predict the potential survival time of GBM patients. Therefore, interactions of miR-323/miR-326/miR-329 and miR-130a/miR-155/miR-210 on survival were initially performed using Kaplan-Meier analysis with the aim at obtaining more specific predictors. The OS curves showed that interactions of two miRNAs were more potent to discriminate survival time of the same patients (Figure 3A). For instance, the OS of patients with low miR-323 and high miR-130a were longer than that of patients with high miR-323 and low miR-130a, with the survival gap of 16.5 months (p = 0.0007; Figure 3 A1). Also, the interaction of high miR-326 and high miR-130a was associated with long OS while that of low miR-326 and low miR-130a was not, with the survival span of 21.5 months (p = 0.0003; Figure 3 A4). Furthermore, PFS analysis showed that several interactions of two miRNAs were more effective to distinguish survival times of patients (Figure 3B). For example, the interaction of high miR-326 and high miR-130a was associated with much longer PFS survival than that of low miR-326 and low miR-130a, with the survival gap of 15.8 months (Figure 3 B2). Collectively, these data indicated that interactions of different levels of miRNAs significantly correlated with OS and/or PFS, and it was potent and effective to discriminate and predict the survival time for GBM patients by this method.

### Functional analyses of the interested miRNAs in GBM

**Figure 4:**
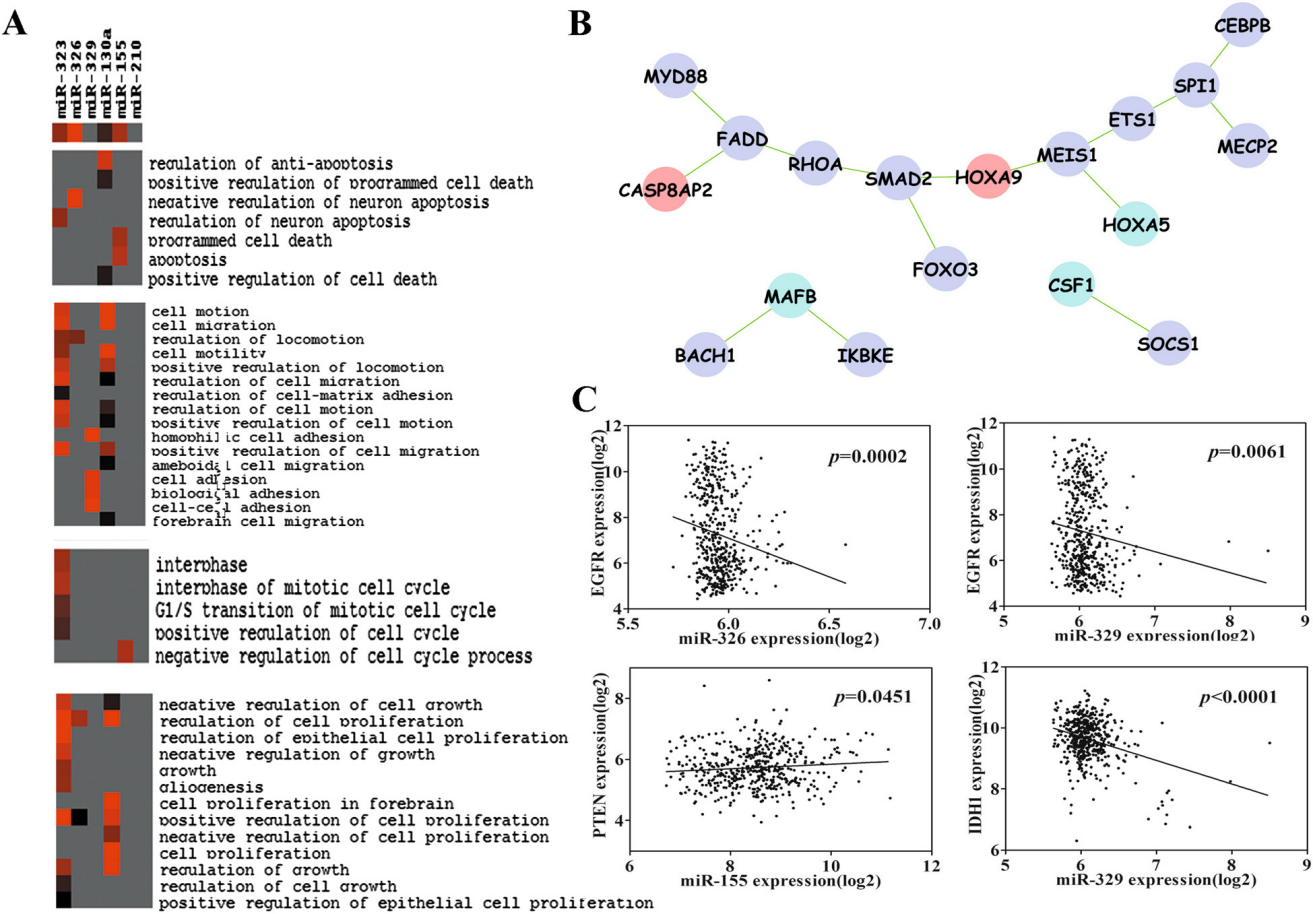
**Functional analysis of the interested miRNAs in GBM. A**. Functional GO biological processes of putative target genes of the 6 miRNAs enriched in cell proliferation and growth, cell cycle, apoptosis and cell adhesion and migration are illustrated. Red denotes highly significant correlation with miRNAs; black denotes lowly significant correlation with miRNAs; grey denotes no correlation. All the GO terms were selected at the criteria of p value < 0.01. **B**. Network of validated target genes of miRNAs. Only the direct interactions between these target genes are exhibited. pp: protein-protein interaction; violet: miR-155 targets; wine red: miR-210 targets; cyan: miR-130a targets. Targets of miR-323, miR-326 and miR-329 are not involved, because of non-interaction among their few validated target genes. **C**. Correlation analysis of the interested miRNAs with confirmed GBM gene markers.

The availability of interaction of miRNAs for prognosis survival was confirmed as above. Subsequently, the rationality was further analyzed. First, GO biological processes of putative targets of miR-323 and miR-130a were enriched in apoptosis, cell proliferation, cell cycle, and cell adhesion/migration, indicating their complicated biological functions during tumor progression, while the enrichment of biological processes in cell adhesion of targets of miR-329 suggested that it mainly took part in tumor migration. Biological processes of target genes of miR-326 and miR-155 were dominantly associated with apoptosis (Figure 4A). Likewise, analysis of KEGG pathway showed that target genes of miR-323, miR-326, miR-130a and miR-155 were all enriched in *Pathways in cancer*, while miR-329 was mainly related with *Adherens junction* (Additional file 2: Table S2). However, there was no enrichment of biological process and KEGG pathway of target genes of miR-210 due to less predictive targets according to TargetScan (Figure 4A and Additional file 2: Table S2). Second, according to Diana TarBase and miRecords, a total of 45 validated target genes of the four miRNAs were observed, whereas the network was built with only 18 real target genes of miR-130a, miR-155 and miR-210, which exhibited direct protein-protein interactions (Figure 4B). This simple net was composed of molecules associated with apoptosis, cell proliferation, inflammation or carcinogenesis, which illustrated that targets of the interested miRNAs were not independent and they interacted with each other. All in all, the complication and cross-talk of the six miRNAs suggested the rationality of interactions of miRNAs to predict the clinical outcome.

Genomic analysis of human GBM showed that EGFR, PTEN and IDH1 were among the most altered genes [23], which are used as commonly monitored markers [24]. Then we tried to determine whether the 6 miRNAs were associated with these confirmed GBM markers, and the analysis showed that miR-326 and miR-329 were negatively correlated with EGFR expression levels, while miR-155 was positively related with PTEN expression levels (Figure 4C). Meanwhile, miR-329 was also inversely associated with IDH1 expression (Figure 4C). These correlation analysis results suggested the potential application of the interested miRNAs in predicting outcome of GBM patients.

### Interactions of miRNAs as prognostic and predictive indicators for survival of patients with GBM

Finally, a univariate Cox proportional hazard regression model was carried out to determine the influence of the 6 miRNAs as well as clinicopathological factors (gender, age and recurrence) on patient survival. This univariate analysis indicated that age, recurrence, expression levels of miR-323, miR-326, miR-329, miR-130a, miR-155 and miR-210 were significantly associated with survival (Table 3). Furthermore, the interactions of miR-323 and miR-130a, miR-326 and miR-155, miR-326 and miR-210, and miR-329 and miR-130 were more sensitively related with survival (Table 3). Then the multivariate Cox regression model was performed to adjust the potentially confounded effects by age, gender and recurrence. The result showed that miR-326, miR-130a, miR-155, miR-210 and the four miRNA-interactions were found to be still significantly associated with survivals, whereas miR-323 and miR-329 had marginal impacts on survival (Table 3). These results indicated that miR-326, miR-130a, miR-155, miR-210 and the 4 miRNA-interactions could serve as prognostic and predictive indicators for GBM patients, which were independent of clinical variables.

**Table 3:** Cox regression analysis of GBM patients in relation to clinicopathological factors and miRNA expression

**Variables**		**Subset**	***P*-Value**	**Hazard ratio**	**95% C.I.**	
					**Lower**	**Upper**
**Univariate analysis**						
Age(year)		20-34/35-64/65+	1.79E-13	2.052	1.695	2.485
Gender		Male/Female	0.801753	0.973	0.787	1.204
Recurrence		Yes/No	8.33E-08	0.530	0.420	0.668
MicroRNAs		Percentile				
Decreased (low/high)	miR-323	75%	0.00430	1.411	1.114	1.786
	miR-326	75%	0.03847	0.770	0.602	0.986
	miR-329	75%	0.01816	1.331	1.050	1.688
Increased (high/low)	miR-130a	25%	0.01028	1.381	1.079	1.766
	miR-155	50%	0.01909	0.778	0.631	0.960
		75%	0.01693	0.744	0.584	0.948
	miR-210	25%	0.00676	0.723	0.571	0.914
		50%	0.00767	0.753	0.611	0.928
Interactions	M323*M130a^@^	75%*25%	7E-05	1.243	1.1165	1.383
	M326*M155	75%*50%	0.00432	0.861	0.778	0.954
	M326*M210	75%*25%	0.00146	0.829	0.739	0.931
	M329*M130	75%*25%	0.00099	1.196	1.075	1.331
**Multivariate analysis**						
Age (year)		20-34/35-64/65+	1.27E-08	1.797	1.469	2.200
Recurrence		Yes/No	9.26E-05	0.620	0.488	0.788
miR-323		75%	0.05391	1.267	0.996	1.611
miR-326		75%	0.00567	0.701	0.544	0.901
miR-329		75%	0.07446	1.244	0.979	1.581
miR-130a		25%	0.00994	1.387	1.082	1.779
miR-155		50%	0.03335	0.796	0.646	0.982
miR-210		25%	0.01697	0.749	0.591	0.949
M323*M130a		75%*25%	0.00142	1.195	1.071	1.333
M326*M155		75%*50%	0.00097	0.840	0.758	0.932
M326*M210		75%*25%	0.00026	0.806	0.718	0.905
M329*M130		75%*25%	0.00342	1.177	1.055	1.313

^@^M denotes miR.

## Discussion

In this study, we identified 38 differentially expressed miRNAs from the most significantly altered miRNAs using data from TCGA dataset. Kaplan-Meier survival and Cox multivariate proportional hazard model confirmed that the expression of miR-326, miR-130a, miR-155 and miR-210 were correlated with OS and PFS of GBM patients and were verified for the first time as independent predictors for GBM patient survival. More importantly, interactions between miR-323/miR-326/miR-329 and miR-130a/miR-155/miR-210 were also significantly related with clinical outcome and were more sensitive to discriminate and predict survival time of patients. Moreover, interactions of miR-323 and miR-130a, miR-326 and miR-155, miR-326 and miR-210 and of miR-329 and miR-130 were also confirmed as independent prognostic indicators to clinical outcome of GBM patients. In addition, the availability and rationality of these interactions as independent prognostic and predictive indicators were supported by integrated analysis of network, biological processes and correlation analysis with confirmed GBM gene markers. Our results suggest a potential application of miRNA profiles and their interactions in development and improvement of prognostic tools and treatments.

Presently, except that presurgical prognosis relies largely on age and Karnofsky Performance Status (KPS), no convincing prognostic and predictive factors have been prevalent in clinical management of GBM patients, although several prognostic and predictive markers or models have been proposed or developed, such as MGMT promoter methylation [25], BRAF fusions and IDH1 mutations [26], subclassification based on gene expression [2], Immunohistochemical analysis [27] and Volume-Age-KPS (VAK) prognostic model related with MR-imaging [28]. Notably, due to development of molecular and gene profiles, molecular stratification for patients’ outcome are increasingly emphasized, which leads to the extensive investigation and exploration of molecular markers.

MicroRNAs, as a family of small non-coding RNAs which are negatively involved in gene regulations, have been recognized as important intervention targets and predictive tools for several diseases because of the stability and convenience of miRNA detection [29-31]. Actually, several study groups have identified a pool of miRNA signatures for clinical outcome prediction. Through screening expression profiles of 200 miRNAs from 84 astrocytoma samples, miR-106a, miR-181b and miR-21 were identified as diagnostic and prognostic markers in defining the signature of astrocytomas and predicting the post-surgical outcome [6]. In another study including 38 GBM samples, miR-21, miR-181c, miR-195, and miR-196b were associated with survival of GBM patients [32]. Using TCGA dataset with 253 individuals, 23 and 19 miRNAs were defined to be associated with OS and PFS, respectively [7]. Also, in another publication with 222 GBM samples, a risk score, formulated on the basis of expression signatures of 10 miRNAs, was associated with GBM patient survival, which was suggested to predict GBM patient survival [33]. On one hand, all these studies indicated that miRNAs were thoroughly involved in GBM biology and several miRNAs could act as predictive and classified indicators for GBM clinical outcome. On the other hand, one concern has been aroused that all the identified miRNAs were almost totally different among these publications, which may be due to different uses of approaches or pre-selections of target miRNAs and so on. In this study, through calculating, sorting and overlapping mean Z-score values in GBM samples from three separate batches, we obtained the most altered miRNAs, which ensured that these candidate miRNAs were more specific and accurate to distinguish expression differences between GBM and normal brain tissues. Herein, the candidate miRNAs in this article were more convincing and feasible for further potential application in clinical practice.

This study did not follow the conventional training and validation test analysis. However, selection bias was yielded and validation of our findings was supported though corroborations as follows. First of all, all miRNAs were selected from the top most altered and overlapped miRNAs which were sorted according to mean Z-scores originated from 3 independent batches. Then, all expression levels of interested miRNAs have been validated on miRNAMap and other independent miRNA detections, which could be considered as external validations. Furthermore, biological function of the interested miRNAs and their target genes were analyzed, which may directly reflect the roles of miRNAs in tumor progression.

Among the 6 interested miRNAs, miR-326 was reported to inhibit GBM cell growth, whereas miR-155 was shown to promote GBM proliferation [34,35], which could be explained by that the target genes of miR-326 and miR-155 were mostly related with apoptosis (Figure 4A). However, according to our knowledge, there is no study reporting the associations between OS and PFS and miR-326/miR-155, while our result for the first time showed that high level of miR-326 and low level of miR-155 were significantly associated with long OS and PFS. Likewise, we first found that low levels of miR-323 and miR-329 correlated with long OS, and high level of miR-130a and low level of miR-210 were linked with extended either OS or PFS. These survival analyses indicated that miR-326 and miR-130a functioned as tumor suppressors while the others as oncogenes. However, it should be noted that expression levels of miR-323/miR-329 were elevated in no-recurrent and longer TTP patients, which were not consistent with oncogenic roles of miR-323/miR-329. Several reasons may be responsible for this inconsistence. Initially, it has been confirmed that on average one miRNA has approximately 100 target sites, regulating a large fraction of protein-coding genes involving in several biological processes, such as cell proliferation, apoptosis, and cell motion etc. [36]. Second, putative targets of miR-323 and miR-329 incorporated a family of molecules associated with cell migration and adhesion as shown in Figure 4A. Furthermore, the recurrence of GBM is related with these migration and adhesion genes [37]. Herein, miR-323/miR-329 may be involved in migration inhibition in non-recurrent patients through elevation of their expression levels. This inconsistence also occurred to miR-130a, which was shown to not only inhibit tumor suppressor RUNX3 in hepatocellular carcinoma [38] but also suppress proto-oncogene MET in lung cancer. This may be due to extensive distribution of predictive targets of miR-130a [39]. To date, there is no functional study related with miR-323, miR-329, miR-130a and miR-210 in GBM.

The complication of biological function of these miRNAs also indicated that it may be more reasonable to study their interactions, because of the multifactorial nature of the disease, and the distinguishing feature of miRNAs that an average miRNA has approximately 100 target sites and regulates a large fraction of protein-coding genes, which form a regulatory network [36,40]. OS and PFS analysis showed that the two-miRNA interaction were more sensitive and accurate to discriminate and predict the survival time in relative to one single miRNA. For instance, the longest gap of mean survival time of OS and PFS occurred on miR-130a, with 8.1 months and 5.4 months, respectively. However, the longest gap of mean survival of OS and PFS was 20.9 months and 15.8 months related with the interaction of miR-326 and miR-130a. Moreover, this interaction effect made the stratification of patients’ survival more detailed and specific. For example, the mean OS of patients with high and low miR-130a was 26.6 months and 18.5 months respectively, whereas the corresponding survival of patients with both low miR-323 and miR-130a was 30.9months, and that with both low miR-323 and high miR-130a was 14.4 months. Therefore, the interaction analysis of miRNAs may provide new views on diagnosis and prognosis of GBM patients.

In summary, we identify miR-326, miR-130a, miR-155 and miR-210 markers related with survival of GBM. More importantly, we determine the availability and rationality of 4 miRNA-interactions as more specific and accurate prognostic and predictive indicators to clinical outcome of GBM patients, implying the application for diagnostic and prognostic tools and treatments.

## Competing interests

All authors declared no conflicts of interest.

## Authors’ contributions

QSW designed the study, carried out data analysis and drafted the manuscript. LS performed bioinformatics analysis. HD, FYM and TY participated in the collection and analysis of the data. PY conceived of the study and participated in its design and coordination and helped to draft the manuscript. All authors read and approved the final manuscript.

## Supplementary Material

Additional files 1 **Table S1.** Parameter estimates of microRNAs associated with survival time at different quartile stratifications. Click here for file

Additional files 2 **Table S2.** KEGG pathway analysis of putative targets of candidate miRNAs. Click here for file
