# Supplementary material for: Interactions of miR-323/miR-326/miR-329 and miR-130a/miR-155/miR-210 as prognostic indicators for clinical outcome of glioblastoma patients
Source: J Transl Med. 2013 Jan 9;11:10. doi: 10.1186/1479-5876-11-10 (PMC3551827; doi:10.1186/1479-5876-11-10)
Supplement: Additional files 1 — Table S1. Parameter estimates of microRNAs associated with survival time at different quartile stratifications. [file 1479-5876-11-10-S1.doc]

**Supplementary Table 1. Parameter estimates of microRNAs associated with survival time at different quartile stratifications.**

| **MiRNAs** | **Quartile** | **Mean** | **SE** | **95% CI** | | **Median** | **SE** | **95% CI** | | **P-Value** |
| --- | --- | --- | --- | --- | --- | --- | --- | --- | --- | --- |
|  |  |  |  | **Lower** | **Upper** |  |  | **Lower** | **Upper** |  |
| miR124a | 75% | 21.8 | 1.4 | 18.9 | 24.6 | 15.1 | 0.5 | 14.1 | 16.1 | **0.0438** |
|  |  | 17.2 | 1.8 | 13.6 | 20.8 | 13.1 | 0.6 | 12.0 | 14.2 |  |
| miR128b | 75% | 18.7 | 1.1 | 16.6 | 20.9 | 14.3 | 0.7 | 13.0 | 15.6 | **0.0241** |
|  |  | 26.7 | 3.4 | 20.1 | 33.3 | 15.9 | 1.3 | 13.3 | 18.5 |  |
| miR132 | 25% | 24.0 | 2.3 | 19.4 | 28.6 | 16.0 | 1.4 | 13.3 | 18.6 | **0.0360** |
|  |  | 19.5 | 1.4 | 16.8 | 22.2 | 14.2 | 0.7 | 12.9 | 15.5 |  |
| miR218 | 75% | 21.6 | 1.4 | 18.7 | 24.4 | 15.2 | 0.5 | 14.2 | 16.2 | **0.0611** |
|  |  | 18.0 | 1.9 | 14.2 | 21.8 | 12.8 | 0.6 | 11.5 | 14.0 |  |
| miR323 | 50% | 23.3 | 1.9 | 19.6 | 27.0 | 15.3 | 0.6 | 14.2 | 16.3 | **0.0148** |
|  |  | 18.3 | 1.5 | 15.3 | 21.3 | 13.1 | 0.7 | 11.7 | 14.6 |  |
|  | 75% | 22.5 | 1.5 | 19.5 | 25.4 | 15.1 | 0.6 | 13.9 | 16.3 | **0.0041** |
|  |  | 15.2 | 1.2 | 12.8 | 17.7 | 13.1 | 1.3 | 10.6 | 15.6 |  |
| miR326 | 75% | 18.9 | 1.1 | 16.7 | 21.2 | 14.4 | 0.7 | 13.0 | 15.8 | **0.0377** |
|  |  | 25.4 | 3.0 | 19.4 | 31.3 | 14.9 | 0.8 | 13.4 | 16.4 |  |
| miR329 | 75% | 22.2 | 1.5 | 19.3 | 25.1 | 14.9 | 0.7 | 13.5 | 16.4 | **0.0176** |
|  |  | 15.8 | 1.3 | 13.2 | 18.4 | 13.5 | 1.0 | 11.6 | 15.4 |  |
| miR433 | 50% | 22.5 | 1.8 | 19.0 | 25.9 | 15.6 | 0.8 | 14.0 | 17.2 | **0.0438** |
|  |  | 18.8 | 1.6 | 15.7 | 21.8 | 13.3 | 0.7 | 11.9 | 14.7 |  |
|  | 75% | 21.6 | 1.4 | 18.8 | 24.4 | 15.1 | 0.5 | 14.1 | 16.1 | **0.0815** |
|  |  | 17.1 | 1.8 | 13.6 | 20.6 | 13.1 | 0.4 | 12.4 | 13.9 |  |
| miR106a | 25% | 27.5 | 2.9 | 21.8 | 33.2 | 17.7 | 1.1 | 15.5 | 19.9 | **0.0009** |
|  |  | 18.2 | 1.2 | 15.8 | 20.5 | 14.1 | 0.6 | 12.8 | 15.3 |  |
|  | 50% | 24.4 | 1.9 | 20.7 | 28.2 | 16.2 | 1.0 | 14.2 | 18.2 | **0.0002** |
|  |  | 16.6 | 1.2 | 14.2 | 19.0 | 13.1 | 0.8 | 11.6 | 14.6 |  |
|  | 75% | 21.9 | 1.4 | 19.1 | 24.7 | 15.1 | 0.6 | 14.0 | 16.2 | **0.0294** |
|  |  | 16.4 | 1.6 | 13.3 | 19.5 | 12.9 | 1.0 | 11.0 | 14.7 |  |
| miR106b | 75% | 21.7 | 1.4 | 18.9 | 24.5 | 15.1 | 0.5 | 14.1 | 16.2 | **0.0381** |
|  |  | 17.2 | 1.8 | 13.6 | 20.8 | 12.7 | 0.9 | 10.9 | 14.5 |  |
| miR130a | 25% | 26.6 | 3.2 | 20.3 | 32.9 | 16.1 | 1.3 | 13.5 | 18.7 | **0.0099** |
|  |  | 18.5 | 1.1 | 16.4 | 20.6 | 14.2 | 0.6 | 13.0 | 15.4 |  |
| miR-155 | 25% | 16.3 | 1.2 | 13.9 | 18.8 | 14.2 | 1.0 | 12.3 | 16.1 | **0.0845** |
|  |  | 22.0 | 1.5 | 19.1 | 24.9 | 14.7 | 0.8 | 13.2 | 16.3 |  |
|  | 50% | 17.4 | 1.3 | 14.8 | 20.0 | 14.5 | 0.7 | 13.2 | 15.8 | **0.0187** |
|  |  | 23.7 | 1.9 | 20.0 | 27.4 | 14.9 | 1.3 | 12.4 | 17.4 |  |
|  | 75% | 18.9 | 1.2 | 16.5 | 21.4 | 14.2 | 0.6 | 13.0 | 15.5 | **0.0164** |
|  |  | 25.8 | 2.9 | 20.1 | 31.4 | 16.7 | 1.6 | 13.7 | 19.8 |  |
| miR21 | 25% | 16.7 | 1.6 | 13.6 | 19.8 | 12.9 | 1.0 | 10.9 | 14.9 | **0.0475** |
|  |  | 21.8 | 1.4 | 19.0 | 24.6 | 14.9 | 0.5 | 14.0 | 15.9 |  |
|  | 50% | 18.3 | 1.5 | 15.4 | 21.2 | 14.3 | 0.8 | 12.8 | 15.9 | **0.0570** |
|  |  | 22.8 | 1.8 | 19.3 | 26.3 | 15.1 | 0.7 | 13.7 | 16.4 |  |
|  | 75% | 18.3 | 1.1 | 16.1 | 20.6 | 14.3 | 0.7 | 12.9 | 15.7 | **0.0080** |
|  |  | 26.8 | 3.0 | 20.9 | 32.7 | 15.3 | 1.0 | 13.2 | 17.3 |  |
| miR210 | 25% | 15.8 | 1.5 | 12.8 | 18.8 | 13.1 | 1.3 | 10.5 | 15.7 | **0.0065** |
|  |  | 22.3 | 1.5 | 19.4 | 25.2 | 14.9 | 0.6 | 13.7 | 16.1 |  |
|  | 50% | 17.5 | 1.3 | 14.9 | 20.0 | 14.1 | 1.1 | 11.9 | 16.3 | **0.0074** |
|  |  | 23.6 | 1.9 | 19.9 | 27.4 | 15.0 | 0.7 | 13.7 | 16.4 |  |
| miR605 | 25% | 24.0 | 2.6 | 18.9 | 29.1 | 15.6 | 1.6 | 12.5 | 18.7 | **0.0933** |
|  |  | 19.3 | 1.3 | 16.8 | 21.8 | 14.3 | 0.7 | 13.0 | 15.6 |  |
| miR92 | 25% | 24.6 | 2.6 | 19.4 | 29.7 | 16.3 | 1.3 | 13.7 | 18.9 | **0.0506** |
|  |  | 19.2 | 1.3 | 16.6 | 21.7 | 14.3 | 0.6 | 13.1 | 15.6 |  |
