# Supplementary material for: Interactions of miR-323/miR-326/miR-329 and miR-130a/miR-155/miR-210 as prognostic indicators for clinical outcome of glioblastoma patients
Source: J Transl Med. 2013 Jan 9;11:10. doi: 10.1186/1479-5876-11-10 (PMC3551827; doi:10.1186/1479-5876-11-10)
Supplement: Additional files 2 — Table S2. KEGG pathway analysis of putative targets of candidate miRNAs. [file 1479-5876-11-10-S2.docx]

**Supplementary Table 2: KEGG pathway analysis of putative targets of candidate miRNAs.**

| **microRNA** | **KEGG Category Term** | **Percent of target number (%)** | **P-Value** | **FDR** |
| --- | --- | --- | --- | --- |
| miR-323 | hsa05200:Pathways in cancer | 4.0462 | 0.0012 | - |
|  | hsa04720:Long-term potentiation | 1.5414 | 0.0034 | - |
|  | hsa04360:Axon guidance | 2.1195 | 0.0041 | - |
|  |  |  |  |  |
| miR-326 | hsa04810:Regulation of actin cytoskeleton | 3.4562 | 0.0009 | - |
|  | hsa05218:Melanoma | 1.8433 | 0.0019 | - |
|  | hsa05200:Pathways in cancer | 4.1475 | 0.0033 | - |
|  | hsa04360:Axon guidance | 2.3041 | 0.0049 | - |
|  |  |  |  |  |
| miR-329 | hsa04520:Adherens junction | 2.2951 | 0.0009 | - |
|  | hsa04310:Wnt signaling pathway | 2.6230 | 0.0070 | - |
|  |  |  |  |  |
| miR-130a | hsa04144:Endocytosis | 3.0543 | 0.0000 | 0.0007 |
|  | hsa04350:TGF-beta signaling pathway | 1.9231 | 0.0000 | 0.0037 |
|  | hsa05200:Pathways in cancer | 3.6199 | 0.0002 | - |
|  | hsa05210:Colorectal cancer | 1.4706 | 0.0007 | - |
|  | hsa04310:Wnt signaling pathway | 2.0362 | 0.0009 | - |
|  | hsa04920:Adipocytokine signaling pathway | 1.2443 | 0.0013 | - |
|  | hsa05212:Pancreatic cancer | 1.2443 | 0.0023 | - |
|  | hsa04070:Phosphatidylinositol signaling system | 1.2443 | 0.0028 | - |
|  | hsa04916:Melanogenesis | 1.4706 | 0.0028 | - |
|  | hsa05214:Glioma | 1.1312 | 0.0031 | - |
|  | hsa04114:Oocyte meiosis | 1.4706 | 0.0067 | - |
|  | hsa05220:Chronic myeloid leukemia | 1.1312 | 0.0099 | - |
|  |  |  |  |  |
| miR-155 | hsa04660:T cell receptor signaling pathway | 3.6697 | 0.0000 | 0.0001 |
|  | hsa05200:Pathways in cancer | 5.5046 | 0.0000 | 0.0059 |
|  | hsa04662:B cell receptor signaling pathway | 2.5229 | 0.0000 | 0.0168 |
|  | hsa05210:Colorectal cancer | 2.5229 | 0.0000 | 0.0462 |
|  | hsa04722:Neurotrophin signaling pathway | 2.9817 | 0.0001 | - |
|  | hsa04010:MAPK signaling pathway | 4.3578 | 0.0001 | - |
|  | hsa04150:mTOR signaling pathway | 1.8349 | 0.0003 | - |
|  | hsa05213:Endometrial cancer | 1.8349 | 0.0003 | - |
|  | hsa05215:Prostate cancer | 2.2936 | 0.0004 | - |
|  | hsa05211:Renal cell carcinoma | 1.8349 | 0.0017 | - |
|  | hsa04910:Insulin signaling pathway | 2.5229 | 0.0020 | - |
|  | hsa05220:Chronic myeloid leukemia | 1.8349 | 0.0026 | - |
|  | hsa05221:Acute myeloid leukemia | 1.6055 | 0.0031 | - |
|  | hsa04012:ErbB signaling pathway | 1.8349 | 0.0060 | - |
|  | hsa05212:Pancreatic cancer | 1.6055 | 0.0091 | - |
|  |  |  |  |  |
| miR-210 | no items |  |  |  |
